# Ecology of *Ixodes pacificus* Ticks and Associated Pathogens in the Western United States

**DOI:** 10.3390/pathogens11010089

**Published:** 2022-01-13

**Authors:** Molly McVicar, Isabella Rivera, Jeremiah B. Reyes, Monika Gulia-Nuss

**Affiliations:** Department of Biochemistry and Molecular Biology, University of Nevada, Reno, NV 89557, USA; mmcvicar@nevada.unr.edu (M.M.); bellarivera2021@gmail.com (I.R.); jeremiahbreyes@nevada.unr.edu (J.B.R.)

**Keywords:** ticks, *Ixodes*, *Ixodes pacificus*, tick-borne pathogens, tick ecology, Lyme borreliosis

## Abstract

Lyme disease is the most important vector-borne disease in the United States and is increasing in incidence and geographic range. In the Pacific west, the western black-legged tick, *Ixodes pacificus* Cooley and Kohls, 1943 is an important vector of the causative agent of Lyme disease, the spirochete, *Borrelia burgdorferi*. *Ixodes pacificus* life cycle is expected to be more than a year long, and all three stages (larva, nymph, and adult) overlap in spring. The optimal habitat consists of forest cover, cooler temperatures, and annual precipitation in the range of 200–500 mm. Therefore, the coastal areas of California, Oregon, and Washington are well suited for these ticks. Immature stages commonly parasitize Western fence lizards (*Sceloporus occidentalis*) and gray squirrels (*Sciurus griseus*), while adults often feed on deer mice (*Peromyscus maniculatus*) and black-tailed deer (*Odocoileus h. columbianus*). *Ixodes pacificus* carry several pathogens of human significance, such as *Borrelia burgdorferi*, *Bartonella*, and *Rickettsiales*. These pathogens are maintained in the environment by many hosts, including small mammals, birds, livestock, and domestic animals. Although a great deal of work has been carried out on *Ixodes* ticks and the pathogens they transmit, understanding *I. pacificus* ecology outside California still lags. Additionally, the dynamic vector–host–pathogen system means that new factors will continue to arise and shift the epidemiological patterns within specific areas. Here, we review the ecology of *I. pacificus* and the pathogens this tick is known to carry to identify gaps in our knowledge.

## 1. Introduction

In recent decades, an increase in the emergence rate of vector-borne zoonotic diseases has presented new challenges and threats to public health [1,2]. Several factors, including land use and climate change, contribute to the increase in these infectious zoonotic diseases. Climate change has expanded the range of vector species, bringing them closer to the human populations and altering vector-borne disease dynamics [3]. For instance, an increase in wildfire numbers and intensity in the Western USA has been predicted to increase the movement of large ungulates that may carry ticks to new areas and establish vector populations [4]. Similarly, new pathogens might be associated with changing host ecology and may also alter human disease risk through infection prevalence with pathogens [5,6].

Lyme disease is the most important vector-borne disease in the United States [7]. Lyme disease is caused by an infection with the spirochete, *Borrelia burgdorferi*, transmitted to humans mainly by black-legged ticks. In the eastern and north-central United States, *Ixodes scapularis* Say, 1821 is the primary vector of *B. burgdorferi* and also transmits *B. miyamotoi*, *Anaplasma phagocytophilum*, and *Babesia microti* [8]. In the western United States, *I. pacificus* is the primary vector of these pathogens [9,10,11].

*Ixodes* ticks are three-host ticks. Each developmental stage (larvae, nymph, and adults) has a different seasonal activity and feeds on different hosts. While some *Borrelia* species such as *B. miyamotoi* can be transovarially transmitted, *B. burgdorferi* cannot but can be acquired by ticks either through blood meals from infected hosts or transstadially [12,13,14,15,16]. Humans are accidental hosts and acquire tick bites, usually during outdoor recreational activities. The nymphal stage is critical for human infections because larvae are usually uninfected and acquire pathogens during blood feeding, resulting in transstadially infected nymphs. Nymphs typically go unnoticed by unsuspecting humans due to their small size and shorter feeding periods than adults (approximately five days vs. 8–10 days of the adult) [17]. Seasonal activity and density of potentially infectious tick life stages are thus critical components for tickborne disease risk assessment.

In the western United States, especially in California, where most studies have been conducted, both tick vectors and the *Borrelia* spirochetes species have a greater diversity [18] compared to the eastern United States. Up to 20 different *Ixodes* species have been reported in California. However, only *I. pacificus* is considered the primary vector of *B. burgdorferi* because it commonly bites humans and thus serves as a major bridging vector of *B. burgdorferi* [19]. The other endemic species which preferentially feed on rodents include *I. spinipalpis*, *I. angustus*, *I. neotomae*, and *I. jellisoni* [20]. The passive surveillance suggested that conventionally recognized non-human feeders (*I. spinipalpis* and *I. angustus*) occasionally parasitize humans [21], suggesting a more complex disease transmission cycle in this region.

Currently, only four *Borrelia* species within *B. burgdorferi* sensu lato (*s. l.*) have been found in the northeastern and upper midwestern United States: *B. andersonii*, *B. burgdorferi* sensu stricto (*s. s.*), *B. kurtenbachii*, and *B. mayonii*, with *B. burgdorferi s. s.* representing the dominant species [22,23], whereas, in addition to *B. burgdorferi s. s*, several species within the *B. burgdorferi s. l.* complex, including *B. americana* [24], *B. bissettiae* [25], *B. californensis* [26], and *B. lanei* [27] have been found in the western United States. However, *B. burgdorferi s. s.* is the only species associated with Lyme disease [18].

The prevalence and ecology of Lyme disease in the western United States differ significantly from that in the northeastern and midwestern United States. The overall incidence of Lyme disease in the western United States is 0.2 cases compared to up to 80 cases per 100,000 persons per year in the northeastern and upper midwestern United States [28]. In the Northeast, *B. burgdorferi* is primarily maintained in white-footed mice (*Peromyscus leucopus*) by *I. scapularis* [29]. However, in the west, white-footed mice have a low tick load and low prevalence of *B. burgdorferi* [20]. In contrast, the western gray squirrel (*Sciurus griseus*) and dusky-footed woodrat (*Neotoma fuscipes*) appear to be the predominant reservoir hosts of *B. burgdorferi* [30,31,32].

This review aims to provide a broad overview of the abiotic and biotic factors that influence the distribution and abundance of *I. pacificus* and the transmission dynamics of tickborne pathogens.

## 2. Habitat

**Figure 1:**
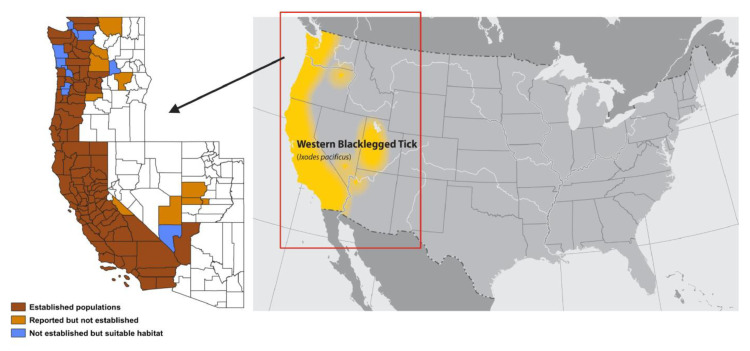
Map of the USA and the Pacific region depicting counties where *I. pacificus* populations are either established, reported, or predicted as suitable by modelling. The yellow color on the US map depicts *I. pacificus* range. Counties in brown have established *I. pacificus* populations. Counties in orange have reported but not established *I. pacificus* populations. Counties in blue do not have an established *I. pacificus* population but are predicted to have suitable habitat by two or more ensemble model members. The USA map was downloaded from the Centers for Disease Control and Prevention website. Background data for this map is adapted from the US National Atlas and published on the National Center for Emerging and Zoonotic Infectious Diseases, Division of Vector-borne diseases. County map is adapted from [33] and Centers for Disease Control and Prevention. Image developed in Biorender.

In the United States, the *I. pacificus* range includes most of the coastal areas of California, Oregon, and Washington, with a few reports from northwestern Utah and Southern Nevada [33]. However, the full range is from British Columbia, Canada, in the North to Mexico in the South. *Ixodes pacificus* is broadly distributed across California [34,35,36,37] and has been collected in 56 of 58 counties [11,31,34,38,39] (Figure 1). The ideal temperature for adult *I. pacificus* ranges from 0 to 20 °C and ideal precipitation between 300 and 600 mm per year; however, habitat suitability increases at precipitation between 200 and 500 mm. A minimum of 40% forest cover and low elevation (sea-level) have been reported as ideal for this species. However, this varies between locations, and *I. pacificus* has been collected from elevation up to 2200 m in northeastern California and Utah [33].

The microclimate, such as the local temperature, wind speed, degree of exposure, soil moisture, tree cover, the direction of the sun, and saturation deficit, can influence tick habitat. The shielded habitats with tree canopy, leaf litter layer, or undergrowth and shrub cover provide a constant microclimate. Dense oak woodlands are the preferred habitat of *I. pacificus* in California. Significantly more ticks were found on trunks of moss-covered oak trees within woodland grass habitats as moss reduces surface temperatures of the tree by an average of 1.9 °C and increases relative humidity (RH) by up to 2.5% [40]. An average of 1.06 ticks was found on all oak trees in northern California, specifically along the coast of Mendocino County; 1.05 ticks on all trees with basal leaf litter, and 1.19 ticks for trees of large circumference were reported [40] supporting the idea that microclimate is important factor for tick abundance.

MacDonald et al. chose three diverse sites in Santa Barbara County, CA, to collect *I. pacificus* ticks weekly from November to June 2013 to 2015. These sites were (1) Coal Oil Point Reserve, a habitat taken over by coastal scrub, grassland, and patches of Coast Live Oak; (2) Paradise Reserve, a habitat dominated by oak woodland with patches of open grassland and interspersed chaparral and scrub; and (3) Sedgwick Reserve, a habitat overrun by oak woodland on northern facing slopes, Oak Savannah in the valleys, and grasslands, chaparral, and scrub [41]. Each site also had varying climates. Coal Oil Point Reserve has a clear marine influence moderating temperatures and providing water subsidies; Sedwick Reserve experiences generally warmer summers and colder winters than the other two sites; Paradise Reserve experiences lower temperature extremes than the other sites Sedgwick Reserve [41]. At Coal Oil Point Reserve, only adult *I. pacificus* ticks were collected. In contrast, at Paradise Reserve and Sedgwick Reserve, all three life stages were collected [41], suggesting that all these habitats are suitable for *I. pacificus*.

Nymphal *I. pacificus* were collected from picnic areas at a similar proportion to leaf litter. In addition, the wooden picnic tables and other wood items, such as tree trunks and logs, also carry *I. pacificus* [42], suggesting that the risk of encountering nymphs around wooded areas is similar to that in leaf litter.

In Utah, Davis et al. surveyed 175 sites and collected 119 *I. pacificus* ticks from six locations between 1700 and 2200 m elevations. Over 90% of the total ticks in Utah were from the Sheeprock Mountains in Tooele County, the habitat characterized by Gambel Oaks, Junipers, Big sagebrushes, Black sagebrushes, and mixed grass [43]. All ticks were collected when daytime temperatures ranging between 6.0 and 29.0 °C [43].

## 3. Tick Distribution

To accurately record *I. pacificus* distributions, a county classification guideline was developed. According to these guidelines, counties may be classified as “established” if either six ticks or two or more life stages are collected in a single year. It is classified as “reported” if the tick collections fail to meet the abovementioned thresholds [11,33]. *Ixodes pacificus* distribution models suggest a potential expansion along the Oregon-Washington border and suitable habitats in 11 additional counties, representing a 12% possible increase in established counties [33] (Figure 1).

In 1998, populations of *I. pacificus* ticks were established in 95 counties in six states, 56 of those counties in California [11], a total of 3.6% of all US counties, whereas these ticks were reported in additional 16 counties (total of 111). Much of the distribution has been on the coast, where the humidity and temperature are higher than the inland [38]. However, the range is slowly expanding [38]. Specifically, the reported *I. pacificus* ticks in Washington were up from five counties in 1998 to six counties in 2015 and established status in Washington increased from 12 in 1996 to 16 counties in 2015 [11]. In total, five counties (Toole, UT; Cowlitz, WA; King, WA; Kitsap, WA; Clallam, WA) now have established population instead of reported or no records, and an additional four counties have reached recorded status for *I. pacificus* (Kittitas, WA; Okanogan, WA; Pacific, WA; Yakima, WA) [11]. Overall ratios of counties classified as “established” versus “reported” increased from 5.1:6 in 1998 to 5.94:1 in 2016, suggesting a modest expansion of *I. pacificus* ticks in western states [11].

The overall abundance of *I. pacificus* ticks in Utah, Oregon, and Washington appears to be low compared to California; however, more work is needed. A distribution map of 2332 *I. pacificus* ticks submitted by Citizen Science Tick Collections from 2016 to 2018 was developed. Out of these 2332 ticks, 54 were from Oregon, 26 were from Washington, and the remaining were collected over 397 Californian sites [44]. In a recent citizen science study [44], 2525 *I. pacificus* ticks (majority adults) were submitted to the Bay Area Lyme Organization and the Arizona State University from 87 counties in California, Oregon, and Washington [44], suggesting higher prevalence in the coastal states. Data collected from other western states is scarce compared to California; increased efforts in tick collections are needed to accurately assess tick distribution in these states.

## 4. Hosts

Access to suitable hosts is critical for the development and survival of ticks. *Ixodes pacificus* feed on a wide range of hosts such as mammals, birds, and reptiles (Table 1). Different host species are preferred depending on the life stage of the tick [45]. The western fence lizard, *Sceloporus occidentalis*, and the southern alligator lizard, *Elgaria multicarinata*, are the most common hosts for immature stages (both larvae and nymphs) of *I. pacificus*. A total of 52 lizards were captured and examined; 162 *I. pacificus* ticks were collected from 8 Southern alligator lizards with larvae to nymph ratio of 3:2; 22 Western fence lizards infested by 323 *I. pacificus* ticks with larvae to nymph ratio of 2:3; 22 Western skinks infested by 35 *I. pacificus* ticks with larvae to nymph ratio of 4:1 [46]. In another study, the western fence lizard accounted for 78% of larval and 98% of nymphal feedings in chaparral in Mendocino County, CA [47], highlighting the importance of lizards as hosts to *I. pacificus* immatures. Eisen et al. suggested that in Mendocino County, CA, immature *I. pacificus* fed significantly on western fence lizards and western gray squirrels (9–35 larvae and 5–6 nymphs per animal). The number of ticks feeding on lizards and squirrels was far greater than that feeding on woodrats, mice, and birds (0.9–3.5 larvae and 0–0.3 nymphs per animal) [48]. Several species of rodents including the dusky-footed woodrat (*Neotoma fuscipes*), the California kangaroo rat (*Dipodomys californicus*), the brush mouse (*Peromyscus boylii*), the deer mouse (*P. maniculatus*), and the pinyon mouse (*P. truei*) have also been shown as *I. pacificus* hosts. The average number of immatures feeding on birds and rodents, 2.9 and 1.3 of 204, respectively, remained low [46]. Out of 182 mammals inspected, three species of rodents (pinyon mouse, brush mouse, and dusky footed woodrat) were infested almost exclusively with larval *I. pacificus* ticks. The deer mouse, *Peromyscus maniculatus*, and Columbian black-tail deer, *Odocoileus h. columbianus*, served as common hosts for adults [46].

Ground-dwelling birds were also common hosts for immature stages in Northern California [45]. Birds found in tick-questing substrates such as leaf litter had higher infestation by *I. pacificus* immature stages (5.2 larvae and 1.0 nymph per bird) than birds that did not utilize or visit leaf litter [48]. A total of 222 birds were collected by Wright et al. and included 19 species, 7 of which (Western tanager, brown-headed cowbird, Cassin’s Vireo, hermit thrush, Pacific-slope flycatcher, acorn woodpecker, and pileated woodpecker) had no ticks [46]. However, the remaining 12 species were infested with *I. pacificus*. Nine species (the Oregon junco, California towhee, spotted towhee, purple finch, black-headed grosbeak, orange-crowned warbler, house wren, brown creeper, and oak titmouse) were infested with both larval and nymphal ticks (Table 1).

Interestingly, American robins and Hutton’s vireo were only infested by nymphal ticks, and one species, lesser goldfinch, had only larval ticks [46]. Hutton’s vireo typically forages high up in trees and is not a common host for ticks. In contrast, the lesser goldfinch forages in densely foliaged trees and shrubs, dry streambeds, and fields and pastures. Chaparral and coastal sage scrub-laden hillsides, including black and white sage, are some of the typical habitats of lesser goldfinch, making it a more suitable host for larval ticks. Bird species that tend to forage in leaf litter or nest on the ground, such as Oregon juncos, Orange-crowned warblers, House wrens, California towhees, and Spotted towhees, were infested with thrice as many larvae than nymphs [46]. In contrast, bird species that foraged on tree bark or canopies such as brown creepers, black-headed grosbeaks, purple finches, and oak titmouse were infested with three times more nymphs than larvae [46].

**Figure 2:**
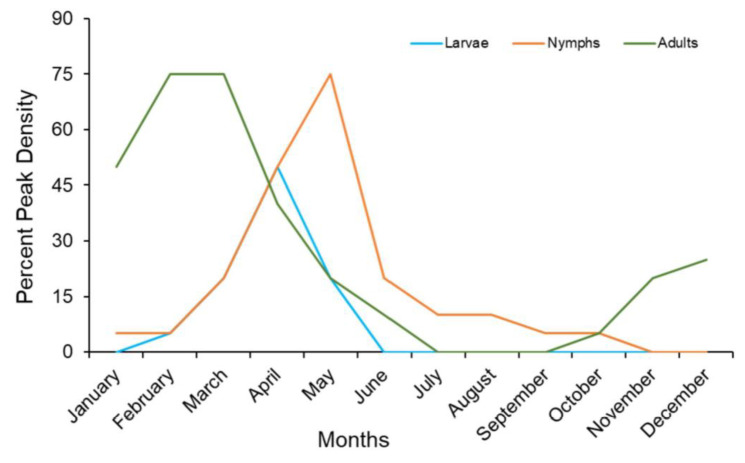
Seasonal density of *I. pacificus* life stages in California. Based on the literature searches, seasonal density was plotted for all three life stages. The numbers are approximation based on all data available on seasonal variations.

Host-seeking behaviors also vary based on location and time of the year. In California, *I. pacificus* populations from the northern and southern parts differ in the times in which they are active. In northern California, larvae and nymphs host-seek from mid-March through late June [49]. In southern California, they start and cease host-seeking earlier, between late February through mid-May and early June [39]. Adult ticks typically engage in host-seeking from November to early May in northern and coastal California. In southern California, host-seeking starts in December and ends by May (Figure 2) [50]. These monthly predictions rely on vegetation, precipitation, and maximum temperature and differ slightly from season to season. While adults are often found ascending vegetation to seek hosts, nymphs and larvae rarely do. Therefore, brushing against the vegetation during outdoor activities leads to the attachment of adult ticks to humans.

## 5. Life Cycle

The lifecycle of *I. pacificus* ticks was closely studied over two decades ago, including oviposition and larval hatching [51]. Replete females were held in individual containers at temperatures ranging between 9 and 29 °C for egg-laying. Females deposited eggs at an average of 9–70 days after drop-off [51]. Egg masses held between 12 and 25 °C hatched approximately 25–178 days after oviposition, while at 9 or 29 °C failed to hatch [51]. The investigators determined that lower temperature thresholds for development, oviposition, and hatching were 6.5 and 9 °C, respectively [51]. During December through March, females collected from Tilden Regional Park in Berkeley, CA, were placed into three habitats (mixed annual grasses, Coyote brush, and grassy slope in between) at the University of California Berkeley Field Station for behavioral research. Berkeley experiences temperature ranges from −1 to 21 °C in winters and 7 to 26 °C in summers. Eggs were deposited between early February and late April, and larvae hatched between early July and late August [51]. The ticks placed in the Coyote brush hatched at an average of 122–158 days following oviposition, while ticks placed in exposed habitats hatched at an average of 130–160 days [51]. Notably, hatching success was lower for eggs deposited in the grassy slope (29%) compared to eggs deposited in the Coyote brush (87%) [51]. All *I. pacificus* larvae from eggs deposited in grassy or exposed habitats died within two weeks of hatching, while all larvae from eggs deposited in brush habitats reported no significant death rates [51]. Of the hatched eggs within the Coyote brush, unfed larvae from 2 of 20 egg masses managed to survive through the winter. They fed readily in late April and then molted throughout August, suggesting that survival during the dry autumn due to brush shelter aided in longer lifespans [51]. Padgett and Lane suggested that *I. pacificus* required more than one year to complete a life cycle that was later confirmed [52].

## 6. Environmental and Climate Factors Associated with Survival

Understanding abiotic and biotic factors and how they may impact the activity, development, and density of *I. pacificus* ticks in specific areas are some of the most complex information to gather. In laboratory studies, the optimum molting rate for *I. pacificus* ticks appears to be 21 °C. A negative correlation was recorded between maximum temperatures and the density of *I. pacificus* nymphs in Sonoma County, California, in 2007, but not in 2006 [53]. Despite this evidence, Swei et al. concluded that habitat variables indirectly affect the relationship between *I. pacificus* tick density and maximum temperatures [53]. 50–75% of nymphal peaks were positively correlated with rainfall and negatively correlated with maximum air temperatures during April and May [54]. *Ixodes pacificus* ticks that were collected from cooler/moister redwood sites, compared to warm/dry oak woodland sites, typically reached 50% of their peak 10–15 days later, remained at 50% of their peak 1.3–1.5 times longer, and started declining 4–6 weeks later [54]. Together, these results suggest that *I. pacificus* nymphal density starts to decline at temperatures between 21 and 23 °C and at an average maximum daily relative humidity (RH) below 83–85% [54].

Areas in California that experience warm and wet winters are reportedly most suitable for *I. pacificus* ticks. More specifically, suitability for *I. pacificus* ticks increases between 0 and 400 mm of cold-season precipitation, a plateau between 400 and 800 mm, and decreases when rainfall exceeds 800 mm. Therefore, the most suitable areas are the ones that receive moderate amounts of cold-season precipitation. In contrast, the least suitable areas are drier or high precipitation regions [55]. Excessive heat between 32 and 40 °C increases rates of tick mortality and decreases rates of successful reproduction [55]. MacDonald et al. suggested that high rainfall may inhibit or reduce host-seeking activity for all life stages. In contrast, high humidity allows for longer intensive host-seeking activity while expending less energy [56]. The same study concluded that hot and dry regions with increasing temperatures are less suitable while wet and cooler areas are better suited for *I. pacificus* survival [56].

The survival rates of immature *I. pacificus* ticks were studied utilizing one laboratory setting and two field sites in northern California: Sonoma County and Quail Ridge Reserve. In all three locations, the survival of larval and nymphal ticks ranged from 90 to 400 days [47]. Relative humidity was at its lowest during the summer seasons in Quail Ridge (74.9%) and Sonoma (76.0%). Although RH remained higher in Sonoma than in Quail ridge, the temperature was higher in Quail Ridge than in Sonoma (21.6 vs. 20.1 °C) [47]. The same pattern was also observed in the winter season [47]. The first purge of ticks occurred at all sites at an RH increase from 80 to 100% and a temperature decrease from 22 to 15 °C; however, the purge also happened within the laboratory setting where temperature and RH ranges remained constant at 22.5–22.9 °C and 70–70.7%, respectively. Thus, fewer *I. pacificus* ticks died at this point within the field than in the controlled setting, and the ticks at Quail Ridge had an overall lower mortality rate than ticks in Sonoma [47]. Although RH remained higher in Sonoma, it might have led ticks to be more active, expending their energy quicker than ticks in Quail Ridge, resulting in decreased survival.

The effects of wildfires on *I. pacificus* populations in California have also been studied to some extent. Increased abundance of questing adult and nymphal *I. pacificus* was reported in the year following a fire and a decrease after that, possibly due to adverse effects on the microclimates caused by the fire [57]. However, another study [58] found no significant change in tick abundance following a wildfire. This vital question remains open as wildfire season increases in duration and overlaps with *I. pacificus* questing season.

## 7. Seasonal Density

Seasonal density can be considered crucial when comprehending the risks regarding concentrated numbers of *I. pacificus* ticks in a specific area. The earliest credible data were acquired over four years from 1998 to 2001 in Mendocino County, California [49]. The nymphal peak reached 50% from early to mid-April in studies from 1998 to 2001, with the average maximum daily air temperature between 15 and 20.5 °C [49]. Nymphal activity exceeding 50–75% of peak densities was positively correlated to rainfall from April to May [49] (Figure 2). Nymphal tick activity started declining at an average temperature of 21 °C. During the initial 20 days of nymphal tick decline, average temperatures were between 23.0 and 28.0 °C during the study period of 1998–2001 [49]. However, the authors concluded that no direct correlations between the rate of decline and temperature were found.

In California from 2012 to 2015, the first *I. pacificus* larvae activity was observed at Sedgwick Reserve in late February, and larvae were absent by mid-May. At Paradise Reserve, larvae were first observed in early March and absent by mid-May, and at Stunt Ranch Reserve, first observed during early to late February and absent by early to late May [39]. Similarly, in Santa Barbara County, CA, larvae were active between March and May, with minimal activity during late January and February [59] (Figure 2).

A number of studies suggest that *I. pacificus* nymphs typically become active in early to late February and reach 50% of their yearly peak density in early- to mid-April [39,49]. Nymphs typically peaked by early May, fell below 50% of their peak densities by early- to mid-June, and were absent by mid-August [39,49,59]. Interestingly, nymphs in Sonoma County remained active from May through August and were found as late as October [31]. From 2012 to 2015, initial nymph activity was observed at Sedgwick Reserve in late February and was absent by mid-April. At Paradise Reserve, nymphs were observed in early March and were absent by mid-May, while at Stunt Ranch Reserve first activity was in early March and remained active through early June [39]. Using a “Week” model, Eisen et al. (2017) could predict the probability of elevated nymphal densities with great accuracy using calendar weeks. They predicted an overall span of 23 weeks with elevated nymphal density with an increase as early as week 8.9 (late February), continuous increase until week 20.4 (mid-late May). Density decreases below “elevated” thresholds by the week of 31.9 (early August) [60] (Figure 2).

*Ixodes pacificus* adults typically become active during late October and early November and are usually reported after the first rainfall or snow of the season [31]. Following their peak in January, the density of adult ticks decreases and is absent by June and July [31]. When the first adults appeared in mid-December, the peak shifted to February and March [39]. In another study in Santa Barbara County, adult activity was observed between November and December until early June [59]. The seasonal pattern suggests that different stages of *I. pacificus* are active year-round. Because most pathogens are acquired by the ticks transstadially, infected ticks (nymphs and adults) could be present year-round and pose a health risk to humans. Therefore, personal protective measures, as discussed later in this review, must be taken all year around.

## 8. Infection Prevalence in *Ixodes pacificus*

### 8.1. Babesia Species

*Babesia* is a genus of intraerythrocytic parasites commonly known as the causative agents of human and animal babesiosis. *Ixodes scapularis* ticks transmit *Babesia* spp. [61], whereas *I. pacificus* ticks are predicted to be vectors for this pathogen, but it has not been experimentally confirmed as of yet. In a single study that collected adults and nymphs of *I. pacificus* from multiple sites in California, 3.0% of total samples were infected with *Babesia odocoilei* [62]. (Table 2). Recently, *B. odocoilei* was identified in two human subjects and was shown to be pathogenic to humans [63]. However, out of these two confirmed cases, one confirmed *I. scapularis* bite, and another patient could not recall a tick bite; therefore, tick species are unknown [63]. It was also revealed that *B. odocoilei* serologically cross-reacts with *Babesia duncani*, an emerging tickborne pathogen (TBP) in Canada and has also been known to be widespread in the continental USA [64]. Although there is substantive evidence that *ixodid* ticks on the west coast (i.e., *Ixodes angustus*, *Ixodes pacificus*, and *Ixodes spinipalpis*) are vectors of *B. duncani*, this has not been yet experimentally confirmed [65]. Depending on the species of *Babesia*, this zoonotic parasite can be transmitted in ticks by transovarial transmission (female to eggs to larvae) and, also, via transstadial transmission (larvae to nymphs to adults). It is important for the clinicians to realize that there are more than two *Babesia* spp. in North America that causes human babesiosis. Many TBPs have been identified in recent decades, and continuing research is needed in this area.

### 8.2. Bartonella Species

*Bartonella* is a genus of bacteria present in many arthropod vectors [66] and infects *I. pacificus* ticks in California. In Santa Clara County, CA, 19.2% of 151 individually tested ticks were positive for *Bartonella.* Additionally, male ticks were more likely to be infected with *Bartonella* than female ticks [66]. Molecular analysis showed a variety of *Bartonella* strains, which were closely related to cattle Bartonella and several known human-pathogenic *Bartonella* species and subspecies: *B. henselae*, *B. quintana*, *B. washoensis*, and *B. vinsonii* subsp. *berkhoffii*, suggesting that *I. pacificus* adults could be a source for *Bartonella* infections in humans [66]. In another study, adult *I. pacificus* were collected from six California counties: El Dorado, Los Angeles, Orange, Santa Cruz, Shasta, and Sonoma. Except for Los Angeles County, ticks from all other counties tested positive for *Bartonella* [67]. Another study conducted in two northern California counties (Sonoma and Yolo) isolated *Bartonella* from ground squirrels (*Otospermophilus beecheyi*) and various other rodent species (*Peromyscus maniculatus*, *P. boylii*, *P. truei* and *Neotoma fuscipes*). Overall, *Bartonella* spp. were isolated from the blood of 71% (32/45) of the ground squirrels and 1/3 (22/66) of the other rodents [68] (Table 2). Ground squirrel isolates were most closely related to *B. washoensis*, whereas the other rodent isolates were closest to *B. vinsonii* subsp. *vinsonii* and *B. vinsonii* subsp. *arupensis*. The overall prevalence of *Bartonella* in rodents from Sonoma (33.3%) was comparable with reports from other regions of North America [69,70,71]. Two species isolated in northern California (*B. vinsonii* subsp. arupensis and *B. washoensis*) have been described as zoonotic pathogens [72,73,74].

### 8.3. Borrelia Species

*Ixodes pacificus* is the vector of *Borrelia burgdorferi*, the causative agent of Lyme disease, the most important tickborne disease in the United States. From 2003 to 2007, *I. pacificus* nymphs were collected from Mendocino, CA, and DNA was extracted for *B. burgdorferi* detection [75]. In total, 47 of the 322 (14.6%) *I. pacificus* ticks were positive for *B. burgdorferi*. Interestingly, the infection prevalence of *B. burgdorferi* was higher in *I. pacificus* nymphs collected from tree trunks instead of logs but higher in logs than leaf litter or grass [75]. In another study, 9.5% of *I. pacificus* nymphs and 1.4% of adults collected in southern California were positive for *B. burgdorferi* [76]. Nymphs tend to have a higher prevalence of *B. burgdorferi* than adult ticks from the same generational cohort [76].

Tick hosts maintain *Borrelia* in the environment, especially small mammals and birds. The order Passeriformes, and more specifically the family Emberizidae, may play a critical role in distributing and maintaining *I. pacificus* and *B. burgdorferi* in California [45]. The western grey squirrel (*Sciurus griseus*) is thought to be an important host in maintaining *B. burgdorferi* s.s. in woodland habitats [77]. Conversely, many lizard species which are commonly parasitized by immature *I. pacificus*, have a serum that is highly lytic to *Borrelia* species, allowing them to eliminate the pathogen and therefore not play a role in maintaining this bacterium in the population [78] (Table 2).

In Napa County in 2008, two out of the 150 (1.3%) adult ticks were infected with *B. burgdorferi*. In 2009, only one of the 130 adult ticks collected tested positive for *B. burgdorferi* [46]. Wright and colleagues (2011) tested *I. pacificus* larvae fed on rodents; 2 out of 215 larvae were positive; however, none were positive in a different cohort. Instead, in this cohort, 3 of 204 *I. pacificus* larvae feeding on bird species tested positive for *B. burgdorferi* [46]. In a recent study from Salkeld et al., 2.9–3.7% *I. pacificus* adults were infected with *B. burgdorferi*, but multiple sites exhibited a much higher prevalence than expected by the confidence intervals (2.3–3.7%). In contrast, nymphal ticks had an infection rate of 3.2–6.2% [79]. Prevalence varied from site to site, and some sites had a much higher prevalence than the average [79].

Out of 288 adults and 67 nymphs, none were positive for *B. burgdorferi* in Southern California [41]. A more recent, broader study was conducted in California, Oregon, and Washington to record pathogen prevalence in *Ixodes* tick species. Out of 381 *I. pacificus* tick samples (adults and nymphs), 302 were from California, and 1.32% were infected with *B. burgdorferi*; 48 were collected in Oregon, and 2.08% of them were infected; and in Washington out of 31 *I. pacificus*, none were positive [21]. In all three states mentioned above, *B. burgdorferi* prevalence in *I. pacificus* nymphs was higher than in adults [21].

Genetic diversity of *B. burgdorferi* in different tick populations and their host community has been observed. In Marin and Sonoma counties, 31 unique *B. burgdorferi* haplotypes were found [80]. Some haplotypes were unique to specific sites, suggesting that genetic drift occurs between multiple separate populations [80]. Furthermore, the frequency of the most common haplotypes (1, 2, and 3) varied depending on the site and year. Mammals sampled from these areas carried the most common haplotypes; however, higher *B. burgdorferi* s.s. diversity in mammals was observed than the ticks.

In addition to *B. burgdorferi*, other species of *Borrelia* are known to be carried by *I. pacificus*; one of those being *B. miyamotoi*. In a study conducted by Salkeld et al., the prevalence of *B. miyamotoi* in adult ticks in coastal central and northern California counties was 1.3% and as high as 3.9% in some areas. In nymphs, the regional *B. miyamotoi* prevalence was 5.1%, with specific locations such as Bolinas Lagoon in Marin County had a much higher prevalence of 17.8% [79]. Other less known species have been reported in *I. pacificus* populations, such as *B. americana* and *B. bissettiae*. *B. americana* was identified in three adults *I. pacificus* while *B. bissettiae* was observed just once [79] (Table 2). These data were aggregated, suggesting the possibility of underrepresenting the actual distribution of pathogens in these populations. In Mendocino County, Mun et al. found 0.7–1.7% prevalence of *B. miyamotoi* s.l. in host-seeking *I. pacificus* [81].

*Borrelia miyamotoi* is considered an emerging causative agent for relapsing fever, and its ability to be transmitted vertically, horizontally, and transstadially may allow it to spread more efficiently [82]. Nymphal infection with *B. miyamotoi* was 45-fold higher than the larvae, while the adult prevalence was 1.8-fold greater than nymphs [82]. Horizontal transmission accounted for >97% of nymphal infections and 43% of adult infections [82].

Although this review has been primarily focused on *I. pacificus*, it is worth noting that *I. spinipalpis*, a non-human biting tick, may also play a role as a vector of *Borrelia* spp. In a recent citizen science study, *Ixodes* ticks were submitted from California, Oregon, and Washington. The infection prevalence of *B. burgdorferi s. l*. and *B. miyamotoi* in *I. pacificus* ticks was 1.31% and 1.05%, respectively, and the prevalence of *B. burgdorferi s. l.* in *I. spinipalpis* ticks was 14.29%. Furthermore, two species within the *B. burgdorferi s. l.* complex were detected in West Coast ticks: *B. burgdorferi s.s.* and *B. lanei*. *Ixodes spinipalpis* had the highest prevalence of *B. lanei* which may indicate a possible public health threat, and the enzootic life cycle and pathogenicity of *B. lanei* warrant further study [21].

### 8.4. Rickettsiales Bacteria

#### 8.4.1. *Rickettsia* Species

Rickettsiaceae is a family of bacteria most commonly transmitted by an arthropod vector, especially ticks. Pathogenic *Rickettsia* species associated with *I. pacificus* have not been recorded yet. However, an endosymbiont rickettsial phylotype, *Rickettsia monacensis* strain Humboldt (formerly known as *Rickettsia* species phylotype G021), and a novel phylotype G022, which is related to a distant spotted fever group, have been identified in *I. pacificus* [83].

Both vertical and transstadial transmission routes of *Rickettsia* pathogens in *I. pacificus* have been confirmed. All *I. pacificus* ticks tested were positive for the phylotype G021 (100% prevalence), a critical component of *I. pacificus*’ microbiome [83]. However, another study suggested that the burden of phylotype G021 significantly differed between collection sites and habitats. The phylotype G021 burden was lower in ticks collected in southern California compared to central and northern California [84]. *Rickettsia* burden also varies by developmental stage; engorged ticks of all life stages have a significantly higher burden of rickettsial phylotype G021 than unfed. However, *Rickettsia* burden was lower in larval ticks than in adults [85]. *Rickettsia* species phylotype G021 can synthesize folate de novo, suggesting that it might benefit the tick [86,87]. However, antibiotic treatment in *I. pacificus* did not affect embryogenesis, oviposition, or egg hatching [88]. While *Rickettsia* endosymbionts may provide nutritional support for *I. pacificus* ticks, they are assumed to be facultative and not required for the development [88].

Prevalence of the novel phenotype G022 in Napa valley, CA, was 2.0% compared to 100% of G021 in the same counties. No significant correlation was found between the burden of the phylotype G021 in the presence and absence of the phylotype G022, suggesting that the presence of these *Rickettsia* species do not interfere with each other [84].

#### 8.4.2. *Anaplasma* Species

*Anaplasma phagocytophilum* is a rickettsial parasite of humans and other animals and is the causative agent of human granulocytic anaplasmosis. Originally, *A. phagocytophilum* was classified as *Ehrlichia equi* and *E. phagocytophila* [89] but *A. phagocytophilum* was reclassified in 2001. Studies suggest that there might be multiple strains of *A. phagocytophilum* present in the environment [90]. In the western USA, *I. pacificus* is a competent vector of *A. phagocytophilum* [91,92]. A total of 2100 rodents, 20 shrews, and one lagomorph, were evaluated for exposure to and infection with *A. phagocytophilum* and infestation with *Ixodes* ticks [93]. The overall seroprevalence was 15.2%, and the highest values were recorded in dusky-footed woodrats, tree squirrels, and chipmunk species [93]. *Anaplasma phagocytophilum* was also detected in lizards and snakes in California. However, experimental inoculation and feeding by ticks on the infected hosts suggested that they do not serve as primary reservoir hosts of this *Rickettsia* [94]. In 2011 and 2012, blood samples and ticks were collected from 349 individual birds from 48 species in Napa and Yolo counties in California to detect *A. phagocytophilum* and *B. burgdorferi* using pathogen-specific PCR. A higher percentage of ticks in Napa County were infected with *A. phagocytophilum* than at the Yolo County site. Blood samples from a golden-crowned sparrow (*Zonotrichia atricapilla*) and a European starling (*Sturnus vulgaris*) were positive for *A. phagocytophilum* DNA at very low levels [95]. In nymphal and adult *I. pacificus*, *A. phagocytophilum* prevalence was 1% and 10%, respectively [95]. The reservoir hosts on which *I. pacificus* feed have implications in the genetic diversity of *A. phagocytophilum* [90]. Most strains detected were genetically distinct and consistent with those found in reservoir hosts, humans, dogs, and horses [90]. *Ixodes pacificus* feeds on larger mammals and many migratory birds that can travel long distances (Table 2) and could lead to rapid, widespread dispersion of *A. phagocytophilum* that may increase human granulocytic anaplasmosis cases.

#### 8.4.3. *Ehrlichia* Species

Pathogens from the genus *Ehrlichia* are the causative agents of two main human infections, human monocytic ehrlichiosis and human granulocytic ehrlichiosis (HGE). *Ixodes pacificus* ticks can carry both pathogens, *E. chaffeensis* and the unnamed species which cause HGE. As mentioned above, *I. pacificus* also carries the strain *E. equi*, the causative agent of equine granulocytic ehrlichiosis (EGE) [96]. Individual *I. pacificus* carrying HGE and the horse pathogen *E. equi* has been reported in northern California [96]. In California, an infection rate of 3.4% and 2.0% for *E. chaffeensis* and the causative agent of HGE, respectively, was noted in *I. pacificus* [97]. Naturally infected *I. pacificus* has been shown to transmit *E. equi* to previously uninfected horses experimentally; however, natural infection in the field has not been seen [97,98]. In addition to the above species, *Rickettsia* of the *E. phagocytophila* genogroup is known to be carried by *Ixodid* ticks. It has been found to infect equines, ruminants, dogs, and humans (Table 2). One study found this pathogen to infect llamas and was transmitted by *I. pacificus* ticks [99].

**Table 2:** Pathogens and reservoir hosts associated with *Ixodes pacificus.* These data are adapted from several publications as indicated in the reference column.

Disease	Pathogen	Reservoir Host	Reference
Babesiosis	*Babesia odocoilei*	Deer species	[61,62,63,64,65]
Bartonellosis	*Bartonella hensele*, *B. quintana*, *B. washoensis*, *B. vinsonii* subsp. berkhoffi	Ground squirrel (*Otospermophilus beecheyi*), Deer mouse (*Peromyscus maniculatus*), Brush mouse (*P.boylii*), Pinyon mouse *(P. truei*), Dusky-footed woodrat (*Neotoma fuscipes*)	[66,67,68,69,70,71,72,73,74]
Lyme Disease (*B. burgdorferi*), Hard Tick Relapsing Fever (*B. miyamotoi*)	*Borrelia burgdorferi*, *B. miyamotoi*, *B. americana*, *B. bissettiae*	Western grey squirrel (*Sciurus griseus*), birds of the order Passeriforms	[21,41,45,46,75,76,77,78,79,80,81,82]
Anaplasmosis	*Anaplasma phagocytophilum*	Dusky-footed woodrat (*Neotoma fuscipes*), Tree squirrels (*Sciurus* sp.), Chipmunk (*Tamias* sp.), Golden-crowned sparrow (*Zonotrichia atricapilla*), European starling (*Sturnus vulgaris*)	[89,90,91,92,93,94]
Human Monocytic Ehrlichosis, Human Granulocytic Ehrlichosis	*Ehrlichia chaffensis*, *E. equi*, *E. phagocytophila*	Deer species	[96,97,98,99]

## 9. Pathogen Coinfection in *I. pacificus*

Coinfection with pathogens has been recorded in both ticks and their hosts. In a study performed in Northern California, authors tested western grey squirrels (*Scirus griseus*), eastern grey squirrels (*S. carolinensis*), an eastern fox squirrel (*S. niger*), Douglas squirrels (*Tamiasciurus douglasii*), and northern flying squirrels (*Glaucomys sabrinus*) for evidence of coinfection. A total of 14% of grey squirrels were infected with *B. burgdorferi* and *A. phagocytophilum* [100]. Coinfection has also been recorded in deer mice, specifically *P. maniculatus*, one of the main hosts for *I. pacificus* [101]. In another study, approximately 1% of *I. pacificus* were coinfected with *B. burgdorferi* and *A. phagocytophilum*, and 0.12% were coinfected with *B. burgdorferi* and *E. chaffeensis* [102]. However, no *I. pacificus* was coinfected with *E. chaffeensis* and *A. phagocytophilum*, suggesting that only specific coinfections may be able to occur naturally [102]. In a study conducted in Washington state, *I. pacificus* ticks were coinfected with *B. burgdorferi* s.s. and *B. miyamotoi*, as well as *Borrelia* spp. and *A. phagocytophilum* [103]. Future studies of pathogen prevalence in *I. pacificus* populations should identify coinfections and understand interactions between different pathogens and their hosts.

## 10. Tick Control

Tick control is crucial in curbing the impact of tickborne diseases (TBDs). While chemical acaricides such as pyrethroids and organophosphates are mainstay of tick control, the potential of botanical acaricides as an adequate repellant has also been assessed [104]. However, resistance is an increasingly pressing issue and additional integrated tick control measures such as landscape management [105], vaccines targeted for humans [106], deer and rodent targeted vaccines [105,107], and personal protection measures (PPM) need to be taken to adequately control tick populations.

Practicing PPM such as staying on trails, applying insect repellent (on skin and clothing), conducting a body check after being outdoors, washing clothes in hot water, and wearing clothing with good coverage (including tucking pants into socks) is essential [108]. For example, in several studies in the Midwest and the northeastern USA, researchers found that people who used insect repellent and performed a thorough body check after being outdoors were less likely to report a TBD diagnosis [108,109,110], suggesting the importance of PPM.

## 11. Conclusions

Expanding tick habitats and consistent identification of new TBP require long-term studies on tick ecology and TBPs epidemiology. Most of our information on the tick–host–pathogen system comes from very few species, namely *I. scapularis* and *I. ricinus*. Therefore, it leads to a bias and does not reflect the complex dynamics of other *Ixodes* ticks and their associated hosts and pathogens. The pacific west region contains various habitat types at varying degrees of suitability for *I. pacificus* and other *Ixodes* species and many vertebrate host species. Most work has been carried out in California due to better vector surveillance and higher incidence of Lyme disease and other TBD cases than in other western states. Tick-borne diseases involve a complex dynamic system of vectors, hosts, and abiotic and biotic factors. To fully understand these systems, interdisciplinary teams with expertise in tick biology, tick genetics and genomics, computational biology, geography, meteorology, veterinary and human health, as well as vector-control districts and public health, need to work together. This review highlights the gaps in knowledge and the research required to have a more comprehensive picture of tick species and TBD dynamics on the west coast.

## Figures and Tables

**Table 1:** Hosts associated with *Ixodes pacificus* life stages. These data are adapted from [45] and a full list with species names is provided in the publication [45].

*Ixodes pacificus* Life Stage	Preferred Hosts		
	Mammals	Birds	Reptiles
Larvae	29	43	8
Nymphs	30	38	8
Adults	29	2	1

## Data Availability

Not applicable.
